# An Imaging Surface Plasmon Resonance Biosensor Assay for the Detection of T-2 Toxin and Masked T-2 Toxin-3-Glucoside in Wheat

**DOI:** 10.3390/toxins10030119

**Published:** 2018-03-10

**Authors:** Md Zakir Hossain, Susan P. McCormick, Chris M. Maragos

**Affiliations:** Mycotoxin Prevention and Applied Microbiology Research Unit, Agricultural Research Service, U.S. Department of Agriculture, Peoria, IL 61604, USA; Md.Zakir.Hossain@ars.usda.gov (M.Z.H.); susan.mccormick@ars.usda.gov (S.P.M.)

**Keywords:** biosensor, iSPR, gold nanoparticle, T-2 toxin, T-2 toxin-3-glucoside, wheat

## Abstract

A sensitive, rapid, and reproducible imaging surface plasmon resonance (iSPR) biosensor assay was developed to detect T-2 toxin and T-2 toxin-3-glucoside (T2-G) in wheat. In this competitive assay, an amplification strategy was used after conjugating a secondary antibody (Ab_2_) with gold nanoparticles. Wheat samples were extracted with a methanol/water mixture (80:20 v/v), then diluted with an equal volume of primary antibody (Ab_1_) for analysis. Matrix-matched calibration curves were prepared to determine T-2 toxin and T2-G. Recovery studies were conducted at three spiking levels in blank wheat. Mean recoveries ranged from 86 to 90%, with relative standard deviations for repeatability (RSDr) of less than 6%. Limits of detection were 1.2 ng/mL of T-2 toxin and 0.9 ng/mL of T2-G, equivalent to their levels in wheat, of 48 and 36 µg/kg, respectively. The developed iSPR assay was rapid and provided enough sensitivity for the monitoring of T-2 toxin/T2-G in wheat. This is the first iSPR assay useful for detecting the “masked” T2-G in wheat.

## 1. Introduction

T-2 toxin is a trichothecene mycotoxin produced by various *Fusarium* species, such as *F. sporotrichioides*, *F. poae*, *F. equiseti*, *F. acuminatum*, and *F. langsethiae;* and by other genera [1,2]. In a worldwide survey conducted by Biomin among 8721 agricultural commodities from 75 countries, T-2 toxin was found in 23% of samples, with an average contamination level of 25 µg/kg [3]. Samples of wheat (*n* = 342) represented 22% of the contaminated samples, with a mean and maximum level of 21 and 163 µg/kg, respectively. Although contamination levels were higher in oat and barley relative to wheat, the higher daily consumption of wheat made it the most important dietary source for T-2 exposure [1]. In the Finnish diet, more than 50% of the T-2 and HT-2 toxin (type A trichothecenes) has been attributed to wheat alone [4]. T-2 toxin affects all animal species, including humans [2]. The Scientific Committee on Food reported that the critical effects of T-2 toxin are general toxicity, hemotoxicity, and immunotoxicity [5]. Historically, T-2 toxin was associated with Alimentary Toxic Aleukia, a condition in humans characterized by sepsis, hemorrhages, and pancytopenia, after the consumption of overwintered moldy grains [1]. T-2 toxin is considered 10-fold more toxic than deoxynivalenol [6].

Mycotoxins can exist in many forms within a commodity. In certain cases, the so-called “free” or “parent” mycotoxins can be modified through chemical or physical means or by metabolism. Among the plant metabolites of mycotoxins are certain derivatives that are not detected by conventional or routine analyses. For this reason, they are often termed “masked” mycotoxins. As part of their defense mechanisms, plants can render toxins more polar through, for example, conjugation with sugars (such as glucose), organic acids, or sulfate [7,8]. There are several reports where masked mycotoxins such as T-2 toxin-3-glucoside, HT-2 toxin-3-glucoside, deoxynivalenol-3-glucoside (DON-3-glucoside), and zearalenone-14-sulfate were detected and identified in different cereal crops [9,10,11,12]. These masked forms pose a potential threat to consumer safety, as they may be hydrolyzed to form the parent toxins during mammalian digestion [7,13]. The FDA has not issued a regulatory guidance for T-2 or HT-2 toxins. However, the European Commission (EC) has recommended maximum levels for these toxins in cereal and cereal products [14]. The recommended limits are 100 µg/kg in unprocessed cereals and 75 µg/kg in cereal products for human consumption. The regulation also emphasized the need for methods of analysis to include masked forms, particularly mono- and diglycosylated conjugates of T-2 toxin. 

**Figure 1:**
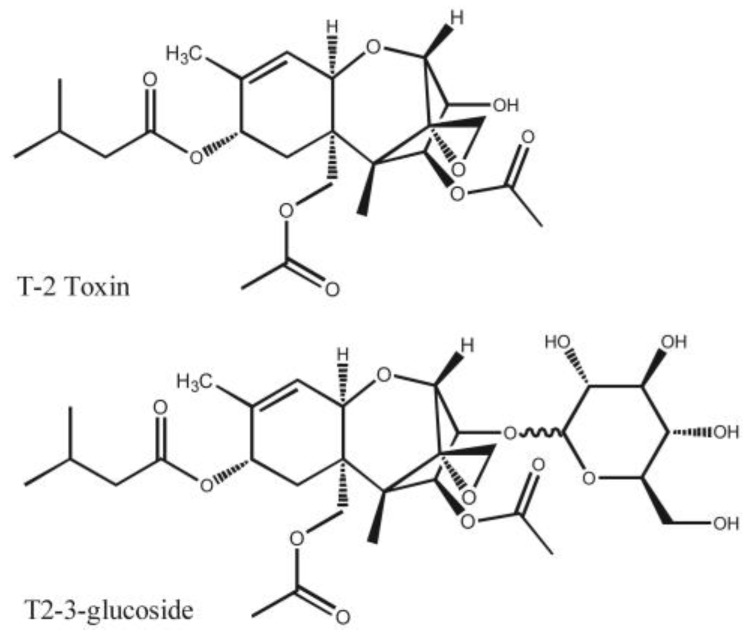
T-2 toxin and T2-3-glucoside (T2-G).

T-2 toxin and T-2 toxin-3-glucoside (T2-G), as shown in Figure 1, have been identified and characterized in wheat and oats, using liquid chromatography coupled to mass spectrometry (LC-MS) [7,10,15,16]. Recently Lattanzio et al. reported an orbitrap mass spectrometric method for the determination of T-2 toxin and T2-G in naturally contaminated barley and malt [13]. LC-MS methods have an advantage in allowing confirmation of the toxins. However, for rapid screening, a simple and less expensive immunochemical method can be a good alternative. Immunochemical methods for detecting masked mycotoxins are still few in number, and are mostly limited to DON-3-glucoside. In addition to chromatographic and traditional immunochemical assays (e.g., enzyme-linked immunosorbent assays or ELISAs, or lateral flow devices), biosensors have become popular tools in the last decade for their industrial applications, particularly in the food sector [1]. Because of the widespread co-occurrence of T2-G and T-2 toxin in naturally contaminated crops, there is a need for a rapid screening assay to monitor for both compounds in food and feed. 

One technology that can be adapted to rapid screening is imaging surface plasmon resonance (iSPR). iSPR is a powerful technique that measures biomolecular interactions in real time. The technique measures changes in refractive index due to the binding of biomolecules to a metallic (often gold) surface. In principle, SPR biosensors use a thin metal (gold/gold spot) film between two transparent media of different refractive indices, such as a prism and a sample solution. SPR occurs when plane-polarized light hits an electrically conducting surface at the interface of two transparent media, from the side of the medium with the highest refractive index (the prism). This generates electron charge density waves called plasmons, reducing the reflected light intensity at a specific angle of incidence (resonance angle). The change is proportional to the mass on the sensor surface. The sensorgram is a plot of response against time, and displays the real-time progress of the biomolecular interaction.

SPR biosensors are typically used to detect the binding of compounds of medium to large molecular weight, because such materials can produce sufficiently large changes in refractive index [17,18]. By immobilizing antibodies onto a sensor surface, it is possible to directly measure the binding of low molecular weight toxins. However, because of their small size, substantial numbers must bind in order to be observed [19]. An alternative approach is to immobilize a toxin–protein conjugate (antigen) onto the surface. In this case, the binding of toxin-specific (primary) antibody is monitored. The advantage to this format derives from the much larger size of the antibody (~150 KDa) as compared with the toxin (0.4665 KDa), which produces a much larger signal as it attaches to the sensor surface. For this reason, most SPR assays for small molecules such as mycotoxins use an immobilized antigen format (also known as an indirect competitive inhibition format). In this format, there is competition between the free toxin and the immobilized antigen for the limited amounts of the primary (antitoxin) antibodies in the sample. This format is also amenable to amplification. Secondary antibodies with gold nanoparticles attached can enhance the SPR signal by binding to minute amounts of primary antibody on the sensor surface [18]. The use of gold nanoparticles can lower the sensitivity of detection to femtomolar levels [20], and reduce the amounts of primary antibody required. 

The advantages of SPR include rapid and simple cleanup procedures, short analysis times, and reusable sensor chips. The latter helps to reduce the cost per analysis. The imaging capabilities of iSPR give it an advantage over traditional SPR, by allowing multiple binding events on different regions of the sensor surface to be monitored simultaneously (multiplexing). This allows, for example, the measurement of multiple antigen–antibody interactions simultaneously in a single injection. This feature facilitates assay development by speeding up the process of determining the best immobilized antigen and the best antibody, and the optimization of the assay conditions. There are a limited number of studies that have used iSPR for the detection of T-2 and HT-2 toxins in cereal and cereal products [21,22,23]. To our knowledge, none of the iSPR methods that have been developed have incorporated detection of the “masked” T2-G. The aim of the present study was to develop a rapid and sensitive iSPR biosensor assay for the detection of T-2 toxin and T2-G in wheat. To determine whether such a method could be used to differentiate blank wheat from wheat contaminated at levels near the EC guideline No. 519/2014 (100 µg/kg, [24]), we investigated the feasibility of establishing cutoff levels for a screening assay.

## 2. Results and Discussion

**Figure 2:**
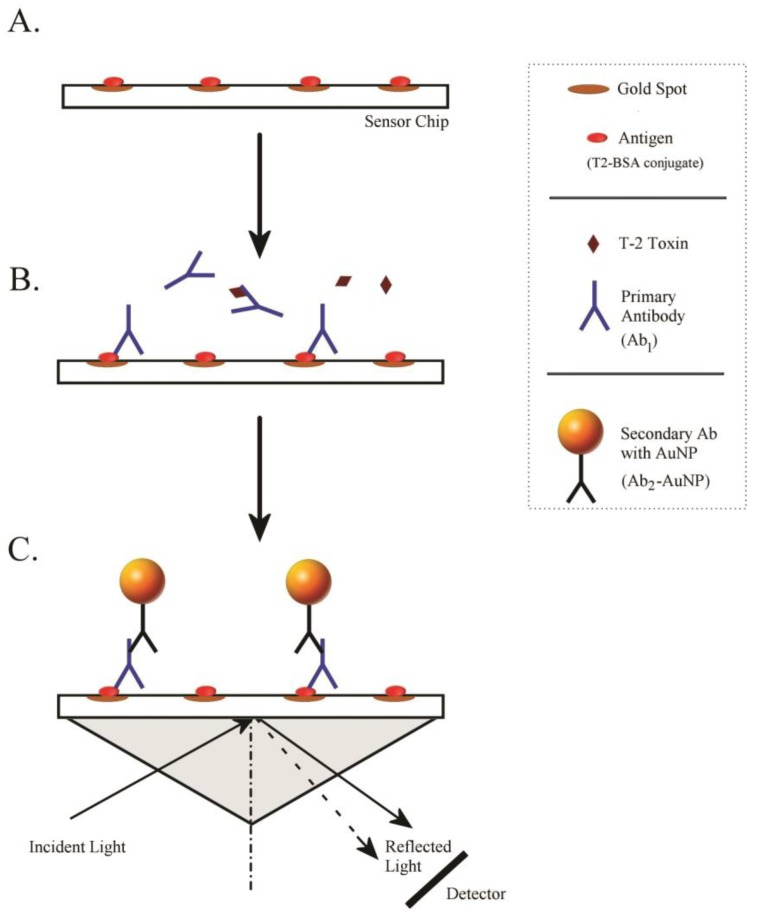
Schematic of the procedure used to detect T-2 toxin and T2-G by iSPR. (**A**) Sensor chip with immobilized antigen; (**B**) Competition between immobilized antigen and toxin for primary antibody; (C) Amplification with secondary antibody-gold nanoparticles (AuNP).

The format for the iSPR biosensor is depicted in Figure 2. The method was based on a competitive immunoassay format, where T-2 toxin was immobilized in the form of T2–protein conjugates (antigens). To provide a sensor surface containing covalently immobilized antigens, the gold surface of the chip was first reacted with carboxylate-containing monothiols. The free carboxylate groups were then activated to NHS esters using EDC and Sulfo-NHS. The primary amines within the T-2 antigens were then reacted with the activated carboxylates, immobilizing the antigens (Figure 2A). When mixtures of samples (containing T-2 toxin or T2-G) and primary antibody (Ab_1_) were introduced, the immobilized antigens competed with the analytes (T-2 toxin or T2-G) for the binding of Ab_1_ (Figure 2B). Ab_1_ that did not bind was removed during flow through the sensor. Following the introduction of the sample and Ab_1_ mixture, a secondary antibody (Ab_2_) labeled with gold nanoparticles (AuNPs) was infused into the sensor (Figure 2C). The Ab_2_–AuNPs were used to increase the amount of material bound to the sensor surface, and thereby amplify the signal over that obtained with Ab_1_ alone. 

### 2.1. Selection of the Optimal T-2 Antigen

**Figure 3:**
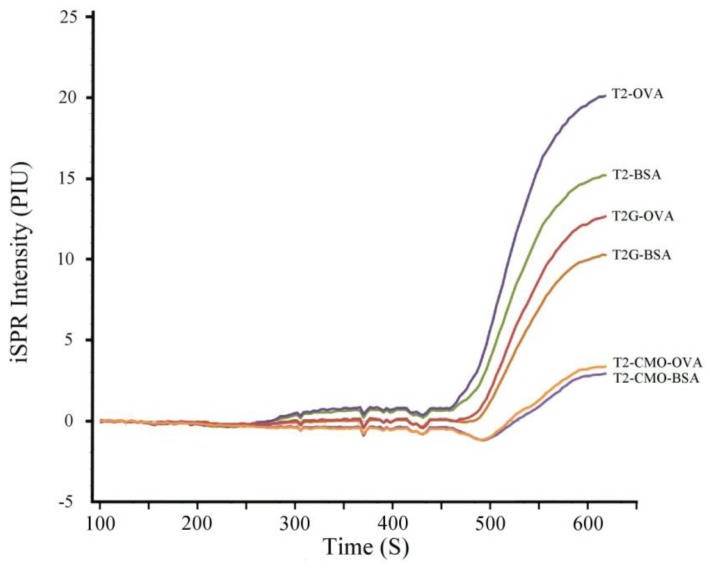
Imaging surface plasmon resonance (iSPR) signals obtained with a single monoclonal antibody (Mab) and six immobilized antigens. This figure shows the raw sensogram data recorded for individual spots coated with the indicated antigens.

To optimize the assay, we investigated six different immobilized antigens containing T-2 toxin or T2-G. The multiplexed nature of the iSPR allows the user to obtain data on multiple antigen interactions in one assay cycle in real time. Figure 3 shows the response of Ab_1_ (monoclonal antibody: Mab 2-13) to each of the six immobilized antigens: T-2–bovine serum albumin (T2–BSA), T-2–ovalbumin (T2–OVA), T-2-glucoside–BSA (T2G–BSA), T2G–OVA, T-2–carboxymethyloxime–BSA (T2–CMO–BSA), and T2–CMO–OVA. Of these antigens, T2–OVA and T2–BSA gave the best iSPR signal intensity, indicating the greatest amount of binding of the Ab_1_ and the Ab_2_–AuNPs. 

### 2.2. Regeneration of Sensor Chip 

A major advantage of iSPR is the ability to reuse the sensor chip. Therefore, in addition to the amount of signal, another factor to consider when selecting the optimal antigen was the number of cycles of use and regeneration that it could withstand. In preliminary experiments, T2–BSA was found to be more stable than T2–OVA (data not shown). The stability of T2–BSA was examined in greater detail using a single Mab (2-13). With T2–BSA immobilized, no significant changes were observed in the baseline and antibody binding capacity after effective regeneration. Over 20 cycles with a single sensor chip (cycle-to-cycle variation) for T2–BSA coated antigen, the iSPR signal intensity or reflectivity was reduced by less than 9% (Figure S1). Spot-to-spot variability was estimated from four replicate spots on the same chip, which yielded a standard deviation of 11%. While the T2–BSA appeared to be stable when immobilized, care must still be taken when handling and storing the sensor chip to prevent loss of coated antigen over the course of time. 

### 2.3. Selection of T-2 Antibody 

While preliminary experiments suggested T2–BSA was the more stable immobilized antigen, the excellent responses observed with immobilized T2–OVA (Figure 3) suggested to us that we should continue to include it in our selection of the best primary antibody (i.e., Ab_1_). This was feasible because the iSPR technology allows for spatial discrimination of the antigens when they are immobilized on different spots of the same chip. Among 10 monoclonal Ab_1_ evaluated on the two immobilized antigens, two antibody/immobilized antigen pairs gave the best responses. These were Mab 1-2 paired with immobilized T2–OVA, and Mab 2-13 paired with immobilized T2–BSA. Previous experience with these antibodies in an ELISA format indicated that Mab 2-13 was the most sensitive for T-2 toxin (IC_50_ = 3.8 ng/mL) and T2-G (IC_50_ = 3.5 ng/mL), and was highly selective for these toxins [25]. Mab 1-2, on the other hand, was the least sensitive by ELISA (IC_50_ = 614 ng/mL for T-2 toxin; IC_50_ = 623 ng/mL for T2-G). Because T2–BSA was the more stable of the two conjugates, and because Mab 2-13 gave the best signal with this antigen, the combination of Mab 2-13 and T2–BSA was selected for further evaluation. 

### 2.4. Effect of Ab_2_–AuNPs on the iSPR Assay 

**Figure 4:**
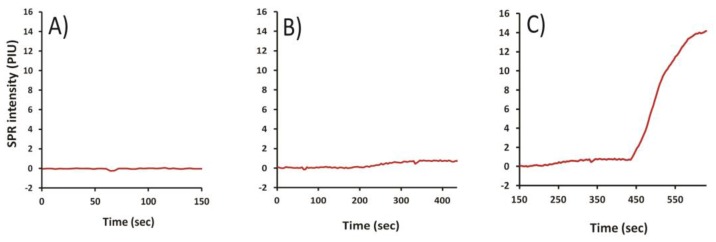
Effect of amplification with secondary antibody labeled with gold nanoparticles (Ab_2_–AuNPs). (**A**) Signal of sensor equilibrated with buffer; (**B**) Effect of the primary (anti-T2) antibody added at 150 s; (**C**) Amplification resulting from addition of Ab_2_–AuNPs at 400 s.

As mycotoxins are small compounds, it is difficult to use iSPR to detect directly their binding to immobilized antibodies. The binding of Ab_1_ causes relatively minor changes in refractive index, as seen in Figure 4. However, labeling of Ab_1_ with Ab_2_–AuNPs can effectively increase the total mass bound. This greater mass significantly changed the refractive index on the sensor chip. The enhancement that resulted from the binding interaction of antigen (T2–BSA) with Ab_1_ (Mab 2-13) and Ab_2_–AuNPs is shown in Figure 4. It was observed with this combination that the signal increased nearly 10-fold when Ab_2_–AuNPs were used, relative to the case where only Ab_1_ was used. This finding closely agreed with that of a previous study, where the signal was enhanced 12-fold when Ab_2_–AuNPs was used [23].

### 2.5. Response to T-2 Toxin and Cross-Reactivity with Analogs

The combination of Mab 2-13 and immobilized T2–BSA gave a reproducible signal, and was used to determine the response of the assay to T-2 toxin and related analogs. As shown in Table 1, the assay exhibited higher cross-reactivity for T2-G than for T-2 toxin. The cross-reactivities of deoxynivalenol (DON), 3-acetyl-DON, 15-acetyl-DON, nivalenol, HT-2 toxin, and HT-2 toxin glucoside relative to T-2 toxin were less than 1%. 

Similar results for cross-reactivity were found with Mab 2-13 when evaluated by competitive indirect ELISA using an immobilized antigen of T2-G (T2G–OVA). In the latter case, the cross-reactivity of DON, 3-acetyl-DON, 15-acetyl-DON, nivalenol, and HT-2 toxin were all less than 3% [25]. Interestingly, the cross-reactivity for T2-G relative to T-2 toxin was greater with iSPR than by ELISA (182% vs 108%). Because the antibody used was the same, we attribute the difference to either the different immobilized antigens or different instrument formats that were used, or a combination thereof.

### 2.6. Assay Performance 

**Figure 5:**
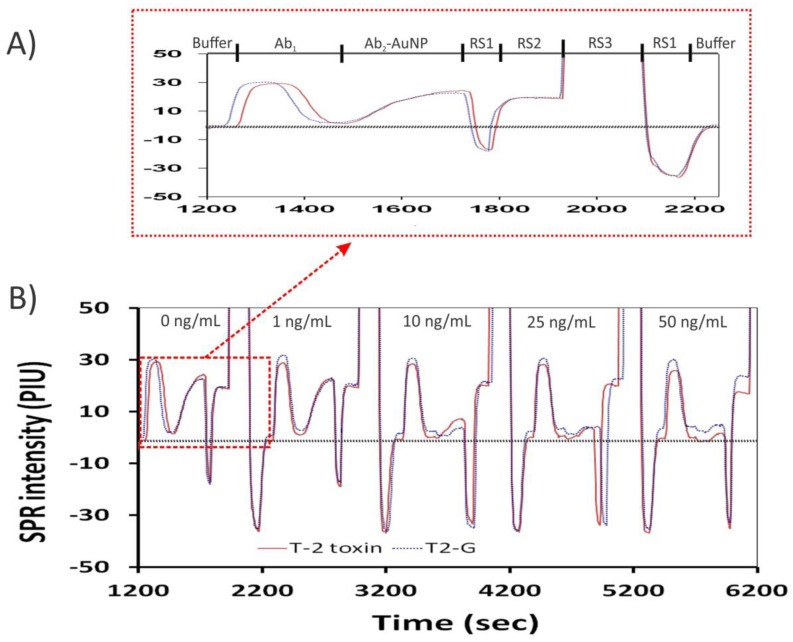
Sensorgrams depicting raw data from five assay cycles in wheat extract. (**A**) Expanded view of a single cycle. Each cycle involved equilibration of the sensor with buffer, addition of primary antibody (and/or toxin) into the wheat extract, addition of Ab_2_–AuNPs, and regeneration with three solutions (RS1, RS2, and RS3, as described in Section 3.6). (**B**) Five cycles, beginning with no added toxin, and demonstrating the effect of increasing levels of T-2 toxin (solid line) or T2-G (dotted line) up to 50 ng/mL. The horizontal dashed line indicates the initial, or zero, signal level. Responses to the toxin were calculated by subtracting the response seen during equilibration from the response seen at the plateau with Ab_2_–AuNPs.

To evaluate the performance of the assay in wheat, samples of blank wheat were spiked, and the measured concentrations of T-2 toxin or T2-G were obtained from matrix-matched calibration curves. The matrix-matched calibration curves were constructed by spiking T-2 toxin or T2-G in extracts of blank wheat at concentrations of up to 50 ng/mL. A sensorgram (Figure 5) shows responses from the sensor over five assay cycles, with increasing concentrations of T-2 toxin present in the spiked extracts. 

The traces shown in Figure 5 are complicated. However, because much of each trace shows the response to the regeneration solutions, only portions of each trace were used to calculate the response to toxin. In each cycle, the initial response coincides with the signal obtained following equilibration with run buffer (generally, near 0). The specific response to the toxin is obtained in the plateau region following addition of the Ab_2_–AuNPs. The difference between the two was inversely proportional to toxin content. For example, in the first cycle, with no toxin, the difference in signal was 23 to 25 PIU, while at 25 ng/mL of toxin the difference in signal was less than 5 PIU. In addition to showing the response to T-2 toxin or T2G, Figure 5 shows the effect of the regeneration solutions (RS1–RS3). The regeneration solutions have refractive indices very different from those of the run buffer, and alternatively cause significant decreases (RS1) or increases (RS2) in sensor response. Importantly, re-equilibration with run buffer caused the sensor response to return to near “0” (the horizontal line in Figure 5), indicating that the regeneration solutions had effectively removed the primary antibody and Ab_2_–AuNPs before the next cycle. Also observed were effects of the bulk matrix during the addition of the primary antibody. These effects occur outside of the regions used for calculating the response to the toxin, and so do not directly interfere.

The working matrix-matched calibration curves for T-2 and T2-G each showed coefficients of determination (r^2^) higher than 0.98. In wheat spiked with T-2 toxin or T2-G, the mean recoveries ranged from 86% to 90%, with average relative standard deviations of repeatability (RSDr) of less than 6% (Table 2). The recoveries for T-2 toxin and T2-G ranged from 80 to 93% and 66 to 102% with RSDr of less than 6 and 12%, respectively. The recoveries and RSDr were within the range of the EC guidelines for T-2 toxin (recovery of 60–130%, RSDr <40%) [14]. The IC_50_ of T-2 toxin and T2-G were 5.17 and 2.84 ng/mL, equivalent to 207 and 114 µg/kg in wheat, respectively. The LOD was 1.2 for T-2 toxin and 0.9 ng/mL for T2-G, equivalent to 48 and 36 µg/kg in wheat, respectively. Recently, Joshi et al. reported that the LOD and IC_50_ of a six-plex nanostructured iSPR assay for T-2 toxin were 25 µg/kg and 580 µg/kg in barley, respectively [22]. While that assay had a lower LOD (25 vs 48 µg/kg), it also had a higher IC_50_ (580 vs 207 µg/kg), indicating that the response to T-2 toxin was less steep than in our investigations. The latter is likely a result of the different antibody/antigen pairs that were used. The IC_50_ of the iSPR assay for T2-G was slightly more sensitive (2.84 vs 3.5 ng/mL) than that previously reported with an ELISA format using the same antibody [25]. The spiking and recovery results suggested that the iSPR assay warranted further evaluation as a possible tool for screening of wheat at regulatory levels.

### 2.7. Establishment of Cutoff Levels for T-2 Toxin

**Figure 6:**
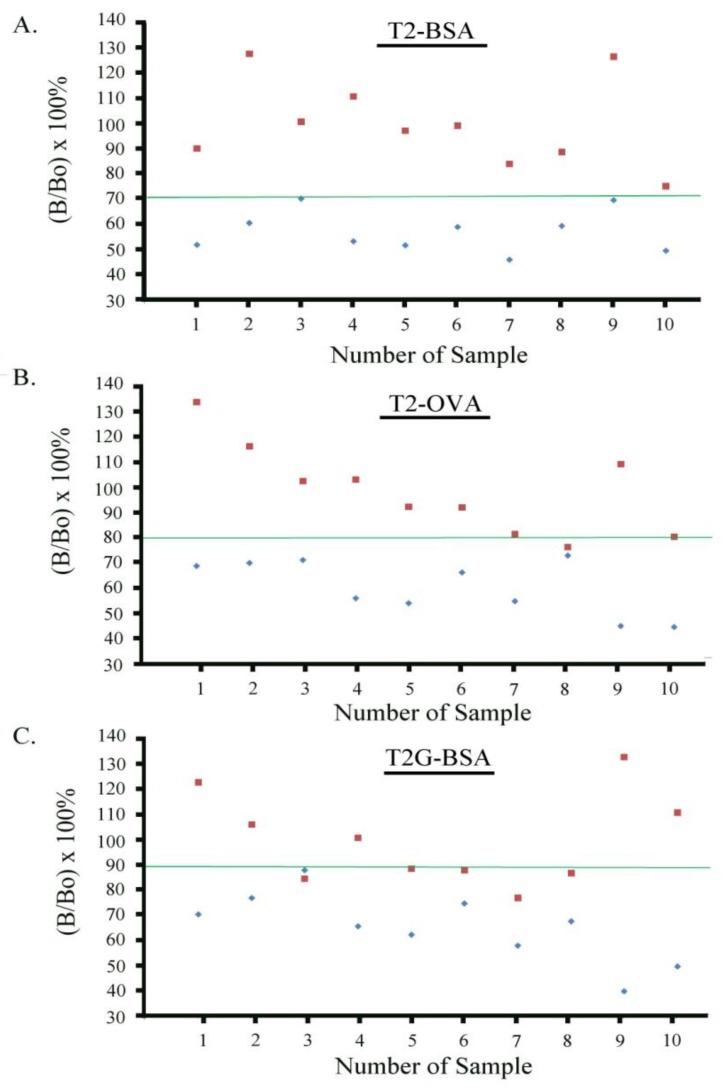
Responses of blank and spiked wheat samples in iSPR assays with three different immobilized antigens; blank samples represented by ♦, and samples spiked at 100 µg/kg T-2 toxin represented by ■. Antigens immobilized were (**A**) T2–BSA; (**B**) T2–OVA; and (**C**) T2G–BSA. The horizontal line (green line) in each panel indicates the cutoff level calculated for T-2 toxin.

To determine whether the iSPR assay could be used as a rapid screening assay based on a cutoff level, we analyzed blank wheat and wheat spiked at the T-2 toxin target level of 100 µg/kg. For comparison, the assays were conducted with the same primary antibody (Mab 2-13) and three different immobilized antigens. The horizontal green line in each panel of Figure 6 shows the cutoff level of T-2 toxin for each of the antigens tested (T2–BSA, T2–OVA and T2G–BSA). For the T2–BSA antigen, it was demonstrated that samples spiked at the target concentration were clearly separated by the calculated cutoff level (71%) from blank samples (Figure 6A). With immobilized T2–BSA, no false positives were observed. However, when T2–OVA and T2G–BSA were used as antigens (Figure 6B,C), it was not possible to clearly separate the target samples from the blank samples at the cutoff level. With the T2–OVA antigen, the false positive rate was over 10%. With the T2G–BSA antigen, the false positive rate was approximately 50%. These results confirmed our preliminary findings that T2–BSA was the optimal antigen when combined with Mab 2-13. This antigen–antibody combination allowed the effective separation of wheat samples containing 100 µg/kg of T-2 toxin from uncontaminated wheat. This finding affirms the applicability of this cutoff level for differentiating samples (blank or contaminated) at the EC target level. 

## 3. Materials and Methods 

### 3.1. Chemicals and Reagents 

Gold (III) chloride trihydrate (HAuCl_4_–3H_2_O) and T-2 toxin (MW: 466.5) were purchased from Sigma Aldrich (St. Louis, MO, USA). T2 toxin-3-glucoside (T2-G; MW: 628.7) was produced at NCAUR-ARS-USDA (Peoria, IL, USA) [26]. Tri(ethylene glycol(EG)_3_ OH-terminated thiol (MW 336.54) was purchased from SensoPath Technologies (Bozeman, MT, USA), and hexa(ethylene glycol)-carboxylic acid (EG)_6_-COOH terminated thiol (MW 526.74) was purchased from Dojindo Molecular Technologies (Rockville, MD, USA). EDC and Sulfo-NHS were purchased from Thermo Scientific (Waltham, MA, USA). Mouse monoclonal antibodies cross-reactive with T-2 toxin (primary antibodies: Ab_1_) were produced at Envigo (Indianapolis, IN, USA) using cell lines developed previously at the USDA-NCAUR [25]. The Mab tested were those designated as 1-2, 1-3, 1-4, 2-5, 2-11, 2-13, 2-16, 2-17, 2-21, and 2-44 in a previous publication [25]. Each of the 10 Mab were used as the primary antibody (Ab_1_) in experiments for selecting the optimal T-2 antigen and antibody combination. 

Calibration curves were used to establish the concentrations of T-2 toxin or analogs required to inhibit the sensor response by 50% (IC_50_). Cross-reactivity of the T-2 antibodies was calculated as: [IC_50_ of T-2 toxin/IC_50_ of analog] × 100%. The cross-reactivity of T-2 antibodies was evaluated against T2-G, DON, 3-acetyl-DON, 15-acetyl-DON, nivalenol, HT-2 toxin, and HT-2 toxin glucoside. Six protein conjugates, T2–OVA, T2–BSA, T2G–OVA, T2G–BSA, T2–CMO–OVA, and T2–CMO–BSA, were used as antigens on the sensor chip. The T2G–OVA and T2G–BSA were produced as described previously, using T2-G and 1,1′-carbonyldiimidazole (CDI) [25]. The CDI-based procedure was originally described by Xiao et al. [27] for linking the 3-hydroxyl group of T-2 toxin to proteins, and was used here to produce the T2–BSA and T2–OVA conjugates. The T2–carboxymethyloxime–BSA (T2–CMO–BSA) and T2–CMO–OVA were produced as described by Zhang et al. [28]. The chemical synthesis of T2–CMO–BSA is shown in the Supplementary Materials (Figure S2). The secondary antibody, goat anti-mouse IgG (Ab_2_), was used for labeling gold nanoparticles (AuNPs), and was purchased from Fisher Scientific (Hampton, NH, USA). The Ab_2_ was noncovalently attached to colloidal gold using the procedure described by Maragos [29]. Run buffer (PBS–BSA) was 0.1% BSA (w/v) in 10 mM sodium phosphate and 0.15 M sodium chloride in water at pH 7.2. Water was prepared using a nanopure water purification system (Thermo Scientific, Waltham, MA, USA). All other chemicals and reagents used in this study were purchased from Fisher Scientific. 

### 3.2. Preparation of Wheat Samples 

The extraction method for T-2 toxin and T2-G in wheat was as described previously [23]. Briefly, 2.5 g of finely ground wheat, prepared with a coffee grinder, was weighed into a 50 mL centrifuge tube and mixed with 10 mL of 80% methanol (in water, v/v). The sample was mixed vigorously for 10 s, and shaken for 30 min on a wrist-action shaker. The samples were kept at 4 °C for 30 min, and then centrifuged at 4000 rpm for 10 min. A subsample for iSPR assay was prepared by diluting 1 mL of the supernatant 5-fold with 0.1% PBS–BSA (run buffer), then centrifuging again at 12,000 rpm for 10 min. Before introducing into the iSPR system, the sample was mixed with an equal volume of Ab_1_ solution. This final solution contained 8% of methanol. 

### 3.3. Preparation of Wheat Samples for the Validation Study 

For T-2 toxin or T2-G determination, the matrix- (blank wheat) matched calibration range of 0 to 25 ng/mL was used with three replicates, equivalent to 0 to 1000 µg/kg in wheat. The limit of detection (LOD) of the assay was defined as the concentration causing 10% inhibition (IC_10_) by T-2 toxin [22]. For the recovery studies, toxins were spiked into blank wheat at three different levels (T-2 toxin: 50, 100, and 500 µg/kg; T2-G: 50, 100, and 200 µg/kg), respectively, in triplicate.

### 3.4. iSPR Biosensor 

An iSPR system (iSPRmager^®^II array system) and SpotReady^TM^ gold coated sensor chips (17 spots) were purchased from GWC technologies (Madison, WI, USA) (Figure S3). The sensor chips were affixed to the optics of the instrument with a refractive index-matching solution. The optical system (CCD camera, Horizon SPRimager) and software (V++ Precision Digital Imaging, ver. 5.0) captured data from all of the manually specified regions of interest (ROI) on the array in real time. 

### 3.5. Optimization of Antigen–Antibody Interaction on the Sensor Chip 

To facilitate covalent attachment of antigens, the gold surface of the sensor chip was modified with a mixture of 0.9 mM EG_3_-OH and 0.2 mM EG_6_-COOH in a total volume of 10 mL of ethanol (99%). To allow sufficient coupling, the solution was held for a minimum of 3 days at ambient temperature in the dark. The free carboxylates on the surface were then activated with an equal mixture of 0.1 M Sulfo-NHS and 0.3 M EDC in deionized water for 1 h. After washing with deionized water and drying with nitrogen gas, the T-2 antigens, T2–BSA, T2–OVA, T2G–BSA, T2G–OVA, T2–CMO–BSA, and T2–CMO–OVA, were spotted (0.065 µg) in triplicate onto the sensor chip. The spotted chips were held in a humid chamber at 4 °C overnight. To reduce nonspecific binding, the surface was blocked with 1 M ethanolamine in 50 mM phosphate buffer at pH 8.5 for 30 min at ambient temperature. After washing with water and drying under N_2_ gas, the antigen coated chips were stored dry at 4 °C until use.

### 3.6. iSPR Assay Conditions

Each assay cycle involved equilibration of the sensor with run buffer, injection of the sample mixed with Ab_1_ (association phase), injection of the Ab_2_–AuNP (amplification phase), and regeneration of the sensor surface by disrupting the antibody–antigen interaction. The optimum dilution (based on iSPR signal intensity) of Ab_1_ (Mab 2-13) was investigated in the range of 1:50–1:20,000, using a stock preparation of 6.7 mg/mL Ab_1_ and dilution in run buffer. The optimal dilution was selected at 1:5000, or 1.34 µg/mL. To start an assay, the sensor surface was equilibrated with run buffer for 150 s. The sample, an extract of wheat containing 8% methanol and mixed with an equal volume of diluted Ab_1_, was then injected into the iSPR system. The flow rate was maintained at 300 µL/min. At 400 s, the labeled Ab_2_–AuNPs were introduced. Subsequently, regeneration was carried out as follows: the regeneration solutions (RS) were introduced in the order of RS1, RS2, RS3, and RS1 (again) at 650 s, 700 s, 850 s, and 1000 s, respectively. The RS consisted of mixtures as follows: RS1: 0.05% Tween 20 in water; RS2: 50 mM sodium dodecyl sulfate and 20 mM NaOH in water at pH 12.0; RS3: 0.1 M glycine–HCl in deionized water at pH 3.0, mixed 9:1 (v/v) with dimethylformamide. To prepare it for the next cycle, the sensor chip was re-equilibrated with run buffer. The entire cycle took 17.5 min. The effect of amplification by AuNPs was investigated by comparing the signal responses between Ab_1_ with and without added Ab_2_–AuNPs.

### 3.7. Cutoff Level Validation for T-2 Toxin 

The purpose for establishing a cutoff level was to demonstrate the fitness for purpose of the screening assay, and therefore its usefulness for the analysis of suspect samples. A successful screening assay must be able to distinguish between blank and spiked wheat samples at the target concentration level. In this case the target concentration was equal to the EC guidance of 100 µg/kg. The cutoff level is calculated from the response of samples spiked at the target concentration. To evaluate the cutoff level, 10 replicate samples of each of two types were prepared. One type was blank samples containing no added T-2 toxin, and the other type was blank samples spiked at the target concentration of 100 µg/kg (25 µL aliquant from 10 µg/mL in 100% acetonitrile). Spiked wheat samples were held overnight at 4 °C before extraction. The experiment was conducted simultaneously for three types of antigens (T2–BSA, T2–OVA, and T2G–BSA) with one selected Ab_1_ (Mab 2-13). The purpose of using three coated antigens on the sensor chip was to see whether all three antigen–antibody pairs could effectively separate blank and spiked wheat samples. The guidelines from the EC regulation No. 519/2014 were used to determine the cutoff level and false positive rates [24]. A detailed explanation of the concept of cutoff level was originally provided by Lattanzio et al. [30]. For a screening assay, the following equation was used to determine cutoff level:

Cutoff level = R_STC_ + (t-value*SD _STC_)

where R_STC_ was the mean response of the positive (spiked) samples at the target concentration (STC), the t-value was the one-tailed t-value for a rate of false negative results of 5%, and SD was the standard deviation observed with the spiked samples. The sample was considered “screen positive” if the relative response (B/Bo) × 100% was equal to or below the cutoff level. 

### 3.8. Data Analysis

The assay response from a particular sample was calculated as the difference between the baseline response during equilibration and the plateau of the response during the Ab_2_–AuNP incubation. Multiple spots with the same coating antigen (four spots for T2–BSA/OVA, and two spots for T2G–BSA/OVA and T2–CMO–BSA/OVA as shown in Figure S3) were used to calculate an average signal. Calculations based upon raw data were made using Microsoft Excel 2007. For preparation of calibration curves, the raw data were normalized to the signal from toxin free samples, i.e., as %(B/Bo), where B represents the response from the sample, and Bo represents the response from the toxin-free control. Nonlinear regression (four parameter logistic dose–response) curves were used to construct the calibration curves, and to calculate the concentrations of T-2 toxin or T2-G in test samples. For this, Table Curve 2D (version 5.01) software was used. 

## 4. Conclusions

A sensitive and reproducible iSPR assay was developed for the screening of T-2 toxin and the “masked” T2-G in wheat. The multiplexing feature of iSPR was used to select the best antigen from among six candidates, and to select the best antibody from among 10 candidates. The optimal combination was immobilized T2–BSA paired with Mab 2-13. Signals from the sensor chip were increased as much as 10-fold by using Ab_2_–AuNPs as an amplification tag. The responses from the iSPR biosensor were consistent over time, and the sensor chip could be reused for over 20 cycles with a loss of signal of less than 9%. In spiked wheat, the mean recoveries for T-2 toxin were 86 ± 4%. For T2-G, the mean recoveries were 90 ± 6%. For qualitative screening, a cutoff level was validated to differentiate uncontaminated wheat from wheat spiked at the target level of 100 µg/kg T-2 toxin, as set by the EC. Thus, the assay allowed for the monitoring of T-2 toxin or T2-G at levels sufficient to meet the EC standard. Further investigations are needed to determine the prevalence of the natural contamination of T2-G, and to assess the risk that may result from exposure. 

## Figures and Tables

**Table 1:** Cross-reactivities of trichothecenes with monoclonal antibody 2-13 in the iSPR biosensor.

Mycotoxins	IC_50_ (ng/mL)	Cross-Reactivity (%)
T-2 toxin	5.17	100
T2-G	2.84	182
DON	ND ^a^	<1
3-acetyl-DON	ND	<1
15-acetyl-DON	ND	<1
HT-2 toxin	ND	<1
HT-2 toxin glucoside	ND	<1
Nivalenol	ND	<1

^a^ IC_50_ was either greater than 517 ng/mL or could not be determined because the concentrations required to do so were too high.

**Table 2:** Recoveries of T-2 toxin and T2-G from spiked wheat (*n* = 3).

Spiking Levels (µg/kg)	Recovery (%)	
	T-2 Toxin ± RSDr	T2-G ± RSDr
50	84 ± 5%	102 ± 2%
100	80 ± 6%	66 ± 4%
200	---	102 ± 12%
500	93 ± 2%	---
Average	86 ± 4%	90 ± 6%

## Supplementary Materials

The following are available online at http://www.mdpi.com/2072-6651/10/3/119/s1. Figure S1. Durability of a T2-BSA sensor chip over multiple cycles, Figure S2. T2-CMO-BSA synthesis, Figure S3. Sensor chip, showing the immobilization of 6 test antigen and a control spot.

## Abbreviations

The following abbreviations are used in this manuscript:

Ab_1_	primary (antitoxin) antibody
Ab_2_–AuNPs	secondary antibody–gold nanoparticles
CDI	N, N’-carbonyldiimidazole
DON	deoxynivalenol
EC	European Commission
EDC	1-ethyl-3 (3-dimetylaminopropyl) carbodiimide hydrochloride
EG	ethylene glycol
LOD	limit of detection
Mab	monoclonal antibody
PIU	pixel intensity unit
RS	regeneration solution
RSDr	relative standard deviation of repeatability
Sulfo-NHS	sulfo-N-hydroxysuccinimide
T2–BSA	T-2 bovine serum albumin
T2–CMO–BSA	T-2–carboxymethyloxime–BSA
T2G	T-2 glucoside
T2-G	T-2 toxin-3-glucoside
T2–OVA	T-2–ovalbumin
