# Supplementary material for: An Imaging Surface Plasmon Resonance Biosensor Assay for the Detection of T-2 Toxin and Masked T-2 Toxin-3-Glucoside in Wheat
Source: Toxins (Basel). 2018 Mar 10;10(3):119. doi: 10.3390/toxins10030119 (PMC5869407; doi:10.3390/toxins10030119)
Supplement: Supplementary file 1 [file toxins-10-00119-s001.docx]

Supplementary Materials: An Imaging Surface Plasmon Resonance Biosensor Assay for the Detection of T-2 Toxin and Masked T-2 Toxin-3-Glucoside in Wheat

Md Zakir **Hossain**,**** Susan P. **McCormick and** Chris M. **Maragos**


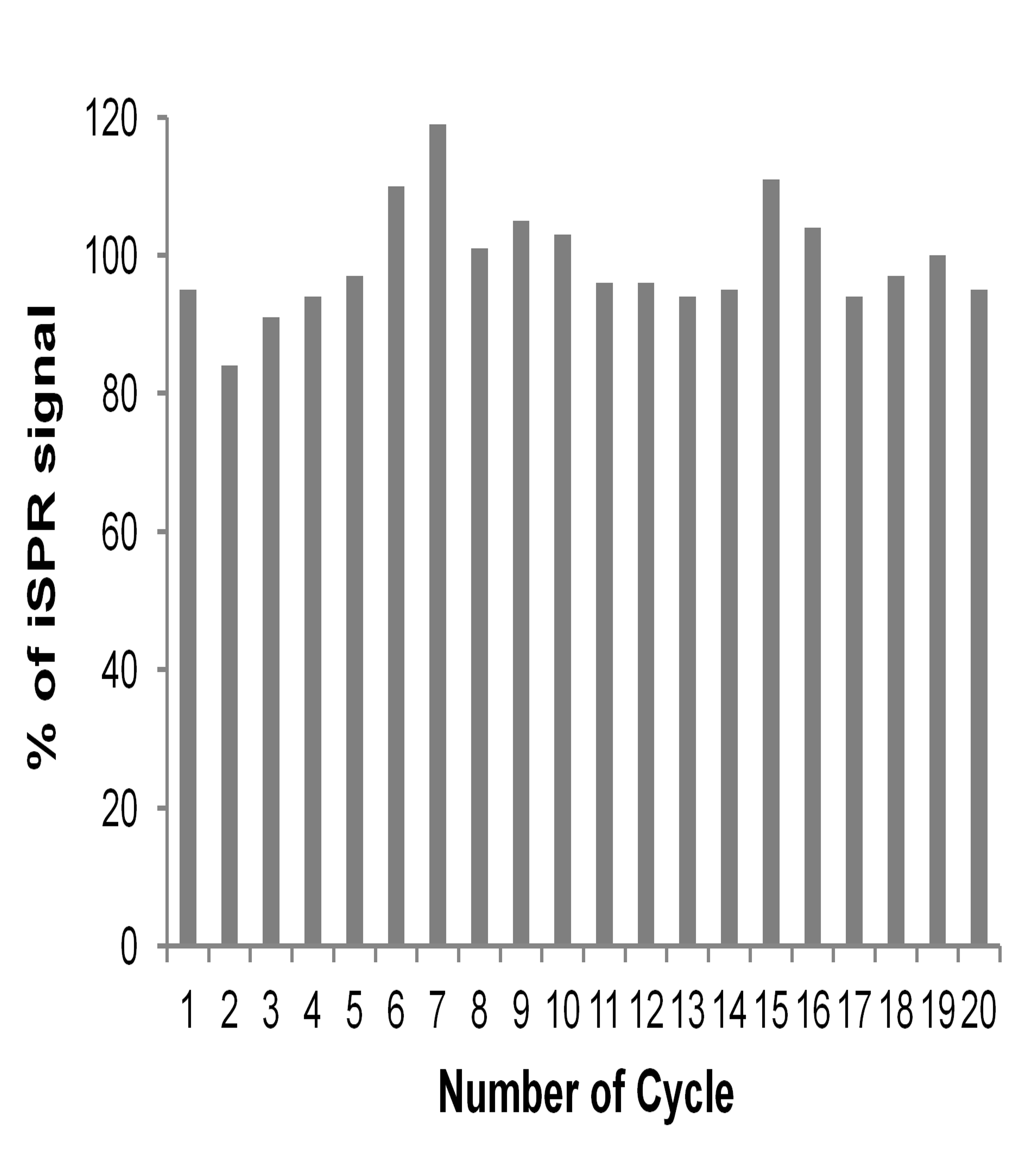


**Figure S1.** Durability of a T2-BSA sensor chip over multiple cycles. Shown are the responses relative to the mean response over 20 cycles (100 ± 9%). Relative responses were calculated by dividing the response from an individual cycle by the mean of all 20 cycles and multiplying by 100%.


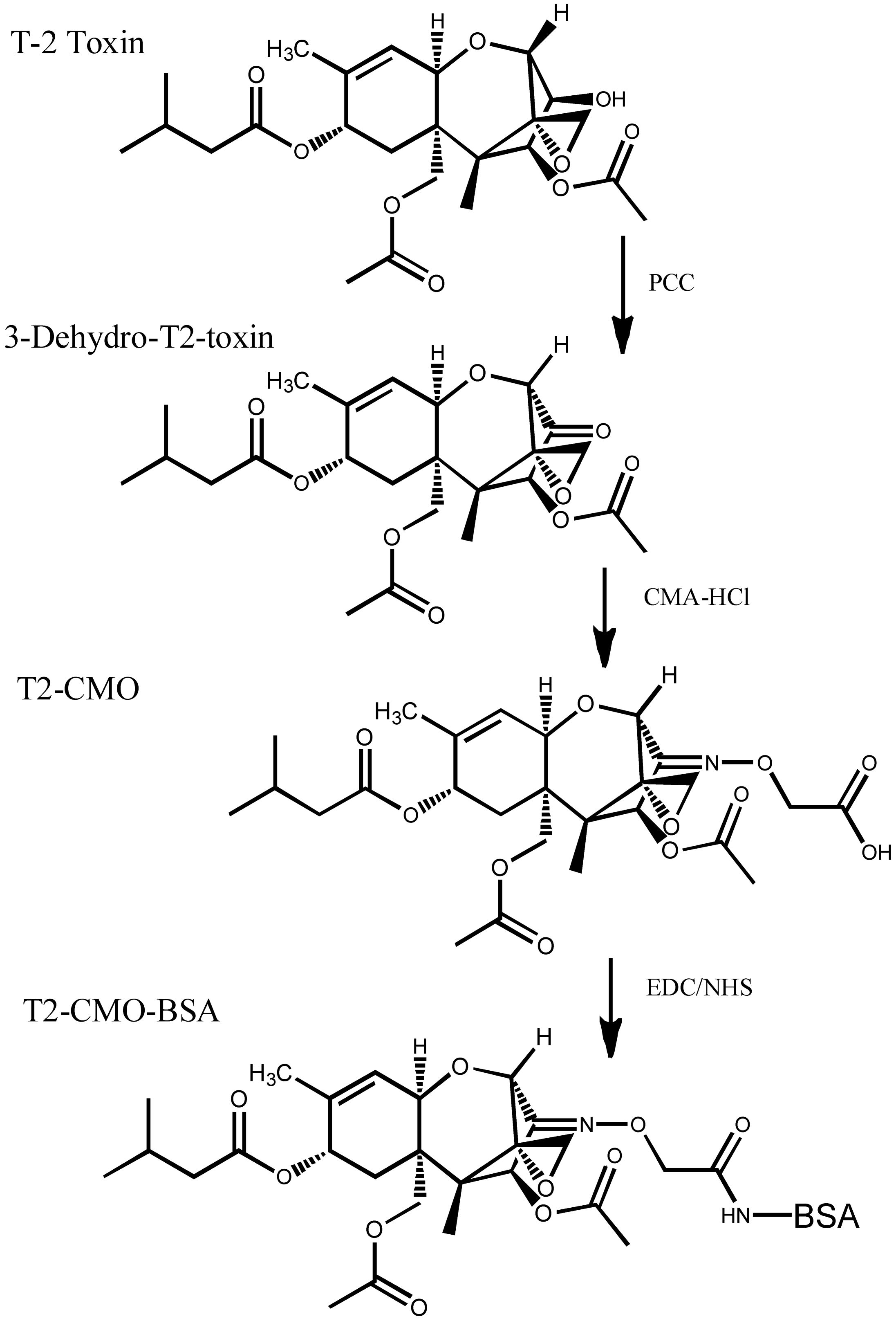


**Figure S2.** T2-CMO-BSA synthesis. The protocol used to synthesize the T2-CMO was that of Zhang et al., [28].


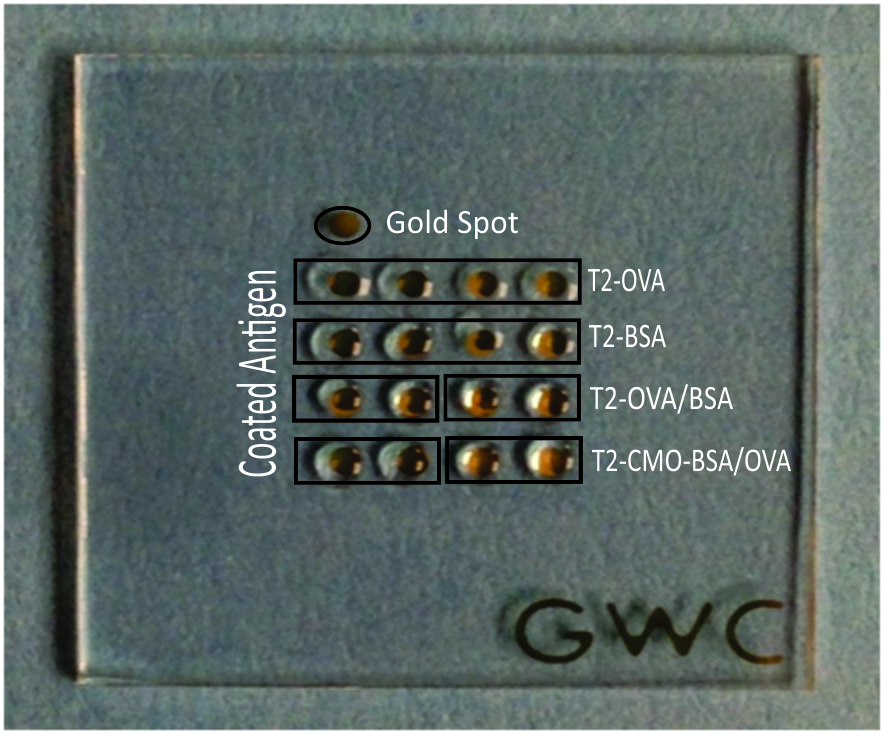


**Figure S3.** Sensor chip, showing the immobilization of 6 test antigen and a control spot.
